# The sticky mittens paradigm: A critical appraisal of current results and explanations

**DOI:** 10.1111/desc.13036

**Published:** 2020-10-05

**Authors:** Linda van den Berg, Gustaf Gredebäck

**Affiliations:** ^1^ Department of Psychology Uppsala University Uppsala Sweden

## Abstract

Almost two decades ago, the sticky mittens paradigm was demonstrated as a way to train reaching and grasping behaviors in pre‐reaching infants, and consequently improve visual attentional abilities. In that first study, Needham and colleagues fitted 3‐month‐old infants with Velcro loop‐covered mittens and allowed them to interact with Velcro hook‐covered toys over the course of 2 weeks. In this review, we scrutinize the 17 studies that have followed those first sticky mittens results in regards to the motor, social perception, and visual attentional domains. Furthermore, we discuss the proposed mechanisms of the sticky mittens training. Current evidence strongly suggests that sticky mittens training facilitates social perception, which is consistent with prior correlational work showing links between action production and action perception. However, studies targeting motor and visual attentional abilities have too diverse results to warrant firm conclusions. We conclude that future research should focus on uncovering if there is a connection between sticky mittens training and motor behavior.

## Graphical abstract

We evaluated the sticky mittens effect in different domains and found that sticky mittens training has the strongest effects in the social perception domain. We emphasize a need for more research in the core domain–the motor domain.

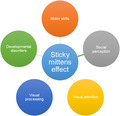

**Research Highlights**

- Sticky mittens are often suggested to enhance manual behaviors, yet the empirical basis for this claim remains unclear.
- Ample support exists for the suggestion that sticky mittens training facilitates social perception.
- Although numerous explanations have been proposed, it is currently unknown what the underlying mechanisms of sticky mittens training are.

## 1 INTRODUCTION

Many studies have suggested that motor training early in life has the ability to elicit reaching and grasping behaviors before they typically would emerge (Lobo & Galloway, 2013; Lobo et al., 2004). Along these lines, Needham et al. (2002) sought to investigate whether young infants with little reaching experiences can be taught to reach and interact with objects with the help of a brief sticky mittens training routine. Three‐ to four‐month‐old infants’ hands were fitted with mittens covered with Velcro loop and provided lightweight toys covered in the corresponding Velcro hook. Over the course of 2 weeks, parents were instructed to put different sets of Velcro toys in front of the infant and to encourage their child to accidentally or intentionally swat the toys for 10 min a day. Contrary to conventional reaching and grasping development, infants obtain an object through a simulated form of both reaching and grasping by mere contact of the mittened hand with the Velcro‐covered object. Following the sticky mittens training, infants’ reaching was assessed relative to a control group that did not receive training. The study demonstrates that infants in the sticky mittens condition performed seven reaches in a 4‐min period, whereas infants in the control condition only performed five reaches on average, illustrating a 40% enhancement in reaching after only 2 weeks of training.

With this study, Needham et al. laid the foundation for a research tradition that connects active exploration through sticky mittens training with enhanced motor behavior, action perception (Gerson & Woodward, 2014a, 2014b; Rakison & Krogh, 2012; Skerry et al., 2013; Sommerville et al., 2005), face encoding (Libertus & Needham, 2011, 2014), and increased visual attention (Libertus et al., 2016) in early infancy. Recent efforts have begun to apply this intriguing idea to infant populations in which motor deficits are prevalent, such as infants born prematurely (Nascimento et al., 2019), those at risk for autism spectrum disorder (ASD; Libertus & Landa, 2014), and those diagnosed with cerebral palsy (Chorna et al., 2015). Sticky mittens training has the potential to bootstrap motor development, visual attention, and cognitive growth through a simple and fun interactive game at an early age. Despite this, a systematic review of the field is lacking. Before reviewing the literature, a short walkthrough of the typical development of reaching and grasping behavior in the first year of life is needed.

## 2 REACHING TO GRASP DEVELOPMENT

At 22 weeks gestation, fetuses exhibit a tendency for goal‐directed manual actions (Zoia et al., 2007), performing different types of manual movements depending on the future endpoint of their hand. After birth, newborns slowly reach their arm out toward objects in front of them, with poor eye‐hand coordination (von Hofsten, 1991). As infants get closer to reaching onset, intense stares, mouthing, and head movements toward the desired object are observed, suggesting a willingness to reach out for objects (Corbetta et al., 2018). The first clear signs of goal‐directed reaching are typically observed between 4 and 6 months of life. At this age, the infant's eye‐hand coordination increases, and they will extend their arm forward and reach, with an open hand, for toys and other interesting items (von Hofsten, 1984; von Hofsten & Rönnqvist, 1988). Six‐month‐old infants reach for moving, and sometimes even occluded, objects in a prospective manner, initiating their reach before an object has entered their reaching space (von Hofsten & Lindhagen, 1979; von Hofsten et al., 1998; Spelke & von Hofsten, 2001). At approximately 7–8 months, infants will learn to adjust their grasp aperture during reach to match the object they are aiming for by prospectively adjusting the hand orientation (Lockman et al., 1984; von Hofsten & Fazel‐Zandy, 1984). Between their first and second birthday, infants will plan their manual actions in multiple steps (Gottwald et al., 2017) and begin to use tools (Kahrs & Lockman, 2014).

Of course, ample individual variation exists in typically developing populations regarding motor abilities (Thelen & Spencer, 1998). Reaching in a dynamic world is an ability that continues to challenge humans throughout life (Hespos et al., 2009). Early motor development is also important for an infant's further development, beyond providing an important interactive interface with the world (Smith & Thelen, 2003). Motor capacities facilitate exploration and learning (Corbetta & Snapp‐Childs, 2009) and have a direct impact on cognition (Gottwald et al., 2016), social understanding (Gredebäck & Falck‐Ytter, 2015), and perception (Schröder et al., 2019).

## 3 THE STICKY MITTENS PARADIGM

Needham and colleagues created the sticky mittens training to examine whether infants’ observation of their own self‐produced reaching actions would boost further development of these manual actions. This contingent feedback was predicted to help infants control and interpret their bodily movements in their environment (Needham, 2016). In the following sections, we provide a summary of the studies that have used the paradigm and discuss the validity and reliability of these findings. This review is thematically structured. We start by discussing the claim that sticky mittens training impacts motor behavior and reaching in young infants, which are the absolute core of the procedure (Needham, 2016). Sections targeting training effects on social perception, visual attention and perception, and its use with clinical or at‐risk populations follow (see also Supplementary Information). We discuss proposed mechanisms of the sticky mittens training and end with a critical discussion about what has been learned and what remains to be proven.

### 3.1 Manual reaching behavior

Libertus and Needham (2010) set out to more closely examine what aspects of this training yielded the previously found facilitating effects on infants’ reaching abilities. As the original study (Needham et al., 2002) used a passive control group, it is difficult to conclude whether the effect is derived from enhanced motor experience reaching for objects, repeated exposure to toys, increased interaction with the parents, or parental encouragement to touch the toys (Libertus & Needham, 2010, 2014; see also Corbetta et al., 2016; Williams et al., 2015). Libertus and Needham (2010) added an additional condition in which parents moved toys around in the infants’ visual field and brought the toys to the infants’ hands in order to provide physical contact with the toy, without allowing the infants first‐hand experience reaching and grasping for the toys. In this review, we refer to this condition as observational, and in this section, all studies had the parent interact with the infant. Parents in both the sticky mittens and observational conditions were instructed to carry out these training routines 10 min a day for 2 weeks, and the results were compared to a group of infants assessed only at post‐test.

Libertus and Needham (2010) tracked changes in reaching abilities using a four‐step reaching assessment. Infants aged approximately 3 months (2.7 months at pre‐test and 3.2 months at post‐test) sat on a parent's lap at a table, facing an experimenter on the opposite side. The experimenter placed an easy‐to‐grasp rattle in four different locations on the table for 30 s in each location. For all infants, the order of placement was identical, starting with the rattle being placed beyond the infant's reach to assess visual attentional capabilities. Subsequently, the rattle was placed within the infant's reach (at the farthest end and then next to the hand), and then in the infant's hands. To assess the behavioral changes before and 2 weeks after training, Libertus and Needham examined the duration of reaching and grasping behavior^1^ during the two steps when the rattle was within the infant's reach. From pre‐ to post‐training assessments, infants from the sticky mittens condition increased their reaching and grasping duration from 12% to 30% of the time. Infants in the observational condition increased their reaching and grasping duration from 5% to 12%, whereas the no training condition (only assessed at post‐test) performed similar behaviors 15% of the time. These results were interpreted in favor of the original suggestion by Needham et al. (2002) that the sticky mittens effect is caused by enhanced reaching experience, not repeated observation of reaching actions. However, the results of this study should be considered with caution, as infants in the sticky mittens condition started out with a higher tendency to reach during the pre‐test. The two groups were not equal to start with. Although these baseline differences were non‐significant, the numerical differences at pretest are rather large. In fact, the sticky mittens group reached twice as much as the control group. This casts doubt on what type of influence this may have had on these findings.

The same authors explored the richness of the sticky mittens training in a study that intended to assess the role of reaching versus encouragement. More specifically, they noted that the original sticky mittens condition was accompanied by encouragement from the parents or experimenter and that this may be another source of the observed enhancement in reaching and grasping (Libertus & Needham, 2014). The rationale behind this follow‐up study was that, when infants learn new motor abilities, they change their social interactions with their parents. Parents will often encourage and praise their child for learning this new ability. For example, when infants learn to walk, parents will praise the child for bringing an object to them or encourage the child to explore the object even further (Karasik et al., 2014). Darcheville et al. (2004) found that, when pre‐reaching movements of 2‐month‐olds are accompanied by praise from their mother, proper reaching behaviors increase. Thus, parent–infant interactions seem to be an important part of the emergence of new motor behaviors. To study this, data from the sticky mittens and observational conditions reported by Libertus and Needham (2010) were reused as a comparison for this new study. Two new training types were added to isolate encouragement and reaching: an encouragement‐only condition and a movement‐only condition. In the former condition, parents were instructed to encourage their child to reach for a toy without physically helping them. In the latter condition, parents were told to attach a toy to their infant's wrist, which allowed the child to act on the toy, without encouraging them to use it. The time infants from the encouragement‐only condition spent reaching for and grasping the toy increased from 11% at pre‐test to 16% at post‐test, whereas this reaching and grasping duration increased from 8% to 13% for infants in the movement‐only condition. This was compared to a change from 12% to 30% in the sticky mittens condition in Libertus and Needham (2010), resulting in the conclusion that neither encouragement nor movement training alone were sufficient to elicit an increase in reaching and grasping behavior (Libertus & Needham, 2014).

A year later, Libertus et al. (2016) followed up on the 2‐week sticky mittens and observational conditions from their previous sticky mittens training study (Libertus & Needham, 2010), when the infants were 15 months old. To assess their current motor behaviors, an additional group of untrained 15‐month‐old infants was recruited as a control group. Interested in the developmental cascades, possibly set in motion by sticky mittens training, they allowed infants to play freely with a bead‐maze toy for 5 min. While looking at the duration of grasping behaviors during this play session, Libertus et al. found that 15‐month‐old infants who earlier took part in sticky mittens training at 3 months of age would grasp and/or hold the toy for 56% of the time. The observational condition and no training condition only grasped the toy for 32% and 28% of the time, respectively. Thus, sticky mittens training still affected grasping behaviors positively 12 months after training. However, caution is warranted, as the original study had inflated pre‐training reaching and grasping values that may have sustained the test of time, and this group may have out‐performed the other groups even without training.

Two additional independent sticky mittens studies have assessed the development of reaching and grasping. Wiesen et al. (2016) conducted a new sticky mittens study (pre‐test at 2.5 months and post‐test at 3 months), including a 2‐month follow‐up at 5 months of age. To assess reaching and grasping behaviors (this study examined reaching and grasping separately) in the sticky mittens and observational conditions, they placed a teething toy in the infant's hands for 15 s, after which they used the four‐step reaching task from Libertus and Needham (2010). In contrast to the previous sticky mittens research, infants did not become more proficient at reaching or grasping post‐test. At the 5‐month assessment, there was still no reaching advantage for the sticky mittens condition. However, the percentage of time the 5‐month‐old infants from the sticky mittens condition spent grasping or holding the toy increased from 4% to 32%, whereas the time spent grasping or holding the toy among infants in the observational condition only increased from 2% to 18%. As this grasping behavior became more pronounced 2 months post‐training, Wiesen et al. (2016) argued that sticky mittens training results in a developmental cascade and enhanced reaching and grasping, with long‐term effects. Around the same time, Needham et al. (2017) demonstrated that a brief training period of 9 min does not appear sufficient to demonstrate motor enhancements in any of the previously used measures (Needham et al., 2017). In this study, 4‐month‐old infants increased the time they spent reaching from 43% to 54%, but this was a non‐significant difference. A change in the opposite direction was found in the brief observational condition in which 4‐month‐olds spent 49% of the time reaching for an object pre‐test and only 28% post‐test. Although this indicates no reaching improvements after 9 min of sticky mittens training, Needham et al. (2017) suggested that this finding may be indicative of a natural habituation to a repeatedly presented object.

Overall, the sticky mittens studies targeting motor proficiency demonstrated weak support for the claim that sticky mittens enhance reaching and grasping. Several factors make it difficult to evaluate the effects of sticky mittens on motor proficiency and skill. Conditions are re‐used in several studies, which limit the number of replications of manual effects. Furthermore, numerical baseline differences could cloud the interpretations of both immediate and long‐term outcomes, as the effects of training cannot be isolated from natural development. The lack of consistency may also stem from the lack of adequate comparison conditions that allow us to disentangle encouragement from simulated reaching and grasping. Moreover, without haptic feedback, infants may not be learning to reach effectively as they would when they learn to reach naturally.

Williams et al. (2015) conducted a study in which they controlled for encouragement but also facilitated haptic feedback for a more natural way of learning to reach. One group took part in a sticky mittens training that facilitated haptic feedback. Another group took part in a non‐sticky mittens training, with tactile feedback, in which they only received repeated exposure to reaching opportunities without toy adherence. Non‐sticky mittens training elicited more reaching behaviors after training compared to the sticky mittens training (Williams et al., 2015). Although these contrasting results may be due to unpleasant experiences with touching Velcro with bare fingers (Needham et al., 2015), additional analyses revealed that contact with Velcro did not hinder object contact during training (Corbetta et al., 2016; Needham et al., 2015). These findings were interpreted to suggest that the stickiness of the mittens may not be crucial for enhanced manual effects.

Two studies have demonstrated an immediate effect post‐test (Libertus & Needham, 2010; Needham et al., 2002), whereas one study demonstrated a delayed effect that emerged only after a few months (Wiesen et al., 2016), and only on a different dependent measure than the one used in the original studies. Two studies did not demonstrate an effect at all (Needham et al., 2017; Williams et al., 2015), though Needham et al. (2017) had a shorter training session. With only five studies investigating the effects on manual behaviors using independently collected data and three showing positive, but diverse, effects (Libertus & Needham, 2010; Needham et al., 2002; Wiesen et al., 2016), additional evidence is required before we make statements about the immediate and long‐term effects of this training, such as those found by Libertus et al. (2016). Paradoxically, even though the support for motor enhancement with sticky mittens is not consolidated, effects on social perception, which are founded on the assumption of a motor training effect, are prominent and consistent. These studies are reviewed next.

### 3.2 Social perception

Numerous studies have suggested that manual abilities are important for perceiving, understanding, and predicting actions performed by others. Learning a specific manual behavior enables infants to predict similar actions performed by others (Gredebäck & Falck‐Ytter, 2015). Six‐month‐old infants who have developed the ability to reach and grasp for objects at 6 months of age are better able to predict the goal of reaching actions performed by others than same‐aged infants who are less proficient reachers (Kanakogi & Itakura, 2011). Woodward (1998) familiarized 6‐month‐old infants with a person reaching for 1 of 2 toys. As most of the studies in this section used this paradigm to assess action understanding, we will describe this in more detail. Following habituation, the two toys switched locations and the person reached for the new toy in the old location or the old toy in a new location. Infants who were able to reach and grasp dis‐habituated to the new goal, even though the movement of the hand was similar, but not to the new movement to the old goal. Together, these studies suggest that a close association exists between manual motor proficiency and action understanding early in life (as reviewed by Gerson & Woodward, 2010; Gredebäck & Falck‐Ytter, 2015). Unfortunately, most of what we know about such action‐social perception couplings in early infancy is based on descriptive or correlational studies. In the early sticky mittens studies that assessed social perception it was believed to be a possible means of providing infants with enhanced manual experience in order to assess the causal relationship between motor ability and social perception (Needham et al., 2002; Sommerville et al., 2005).

Sommerville et al. (2005) was the first to use the sticky mittens paradigm to assess the relationship between reaching and action understanding. Sommerville et al. had 3‐month‐old infants interact with a Velcro hook‐covered teddy bear and ball without wearing mittens to obtain a baseline measurement of infants’ reaching abilities. Directly after this interaction, an experimenter^2^ interacted with the infants during a 200‐s sticky mittens training. Half of the infants did this training prior to habituation, whereas the other half did this training afterward for different purposes. To examine how infants perceived others’ actions, they carried out the same habituation paradigm as Woodward (1998). Following sticky mittens training, infants dis‐habituated to the new goal actions, whereas infants without training before habituation did not exhibit such a difference (Sommerville et al., 2005). In two similar studies, Gerson and Woodward (2014a, 2014b) replicated these findings with independent studies comparing the sticky mittens condition to two control conditions, a yoked observational condition and a no‐training condition. Gerson and Woodward (2014b) further demonstrated that infants are not able to perceive goal‐directed actions if the context changes from sticky mittens training to the subsequent habituation assessment in which people perform reaching actions on novel objects. In this case, the contexts differed in regards to the types of toys used in the sticky mittens training compared to the habituation assessment.

Moreover, Skerry et al. (2013) demonstrated in five experiments that, after training, infants are capable of detecting whether a reaching action is executed efficiently. After sticky mittens, non‐sticky mittens, or no training, infants participated in a habituation study in which a person reaches for an object over an obstacle. Following habituation, infants were presented with the same person reaching for the object without the obstacle being present. The person reached in a similar way as before (i.e., inefficient reaching over an imaginary obstacle) or linearly and clearly goal‐directed. Infants from the sticky mittens condition dis‐habituated to the most efficient route. In contrast, infants from the non‐sticky mittens condition (i.e., their toys would not stick upon contact) and no‐training condition did not seem surprised by the inefficient reaches (Skerry et al., 2013). In a series of five additional experiments, Liu et al. (2019) showed that infants already have the ability to understand the efficiency of reaching, even in the absence of sticky mittens training. However, a meta‐analysis of these experiments without training and the previous five experiments with training (Skerry et al., 2013) indicated that the magnitude of these effects was higher when infants had gone through the sticky mittens training. This further illustrates the importance of sticky mittens training in facilitating an understanding of efficient actions.

Bakker et al. (2016) looked into the neural correlates of the social perception processes impacted by the sticky mittens training and found that its effects go beyond behavioral and social perceptive improvements. While infants watched images of a grasping hand reaching toward or away from an object (depicting a movement congruent or incongruent with the object), their neuronal responses were recorded by an electroencephalography system. Prior to watching these actions, half of the infants had taken part in sticky mittens training and the other half had only observed the experimenter move the toys around and bring them to their hand. Sticky mittens training elicited changes to their social processing on a neural level. These infants had higher amplitudes of the P400 ERP (for a review see Gredebäck & Daum, 2015) in posterior temporal areas when the hand reaching toward the object rather than when the hand reaching away from the object was displayed. The P400 during observation of reaching actions (larger amplitude with goal‐directed reaching actions compared to non‐goal‐directed reaching actions) was previously demonstrated to emerge with the onset of infants’ own goal‐directed reaching, sometime between 5 and 6 months, but the same response could here be elicited earlier with the help of sticky mittens training (Bakker et al., 2014). The P400 used to assess the neural correlates of social perception has previously been used to assess the perception of goal‐directed pointing (Gredebäck et al., 2010), ‘give‐me’ gestures (Bakker et al., 2015), pro‐social (Gredebäck et al., 2015) and chasing (Galazka et al., 2016) interactions, gaze direction (Senju et al., 2006), and biological motion detection (Reid et al., 2008). The results from Bakker et al. (2016) suggest that sticky mittens training facilitates, at least temporarily, the neural circuitry responsible for encoding goal‐directed actions, making the neural activity of 4‐month‐olds similar to what is typically seen in 6‐month‐olds.

In contrast to the work on the effects on manual abilities, six independent studies (i.e., with different samples) have shown direct effects of training on social perception post‐test (Bakker et al., 2016; Gerson & Woodward, 2014a, 2014b; Liu et al., 2019; Skerry et al., 2013; Sommerville et al., 2005). These effects seem to be more clear and robust than the effects on reaching. More work is needed, for example, targeting the degree to which sticky mittens training generalizes to new contexts and the degree to which there is a long‐term effect on social perception. In the next section, we will discuss how sticky mittens training impacts visual attentional abilities and cognitive functions outside the social domain.

### 3.3 Visual attention and perception

#### 3.3.1 Visual attention to objects

Being able to manually explore objects provides infants with opportunities to learn about object properties to detect object boundaries (Needham, 2000) and perceive sounds corresponding to certain object movements (Eppler, 1995). Similar effects have been found in relation to more complex perceptual abilities, such as mental rotation. Infants aged 6 months are better able to mentally rotate an object (Möhring & Frick, 2013) and perceive small forms (Schröder et al., 2019) when provided hands‐on experience with the object, but not following observational experience. Other studies argue that infants, at the onset of reaching and grasping, direct visual attention to objects (Corbetta et al., 2014) at the expense of visual attention to caregivers (Fogel et al., 1992). Thus, many attentional and perceptual changes occur once infants become able to manually explore the world (for similar arguments with respect to the onset of walking and crawling see Campos et al., 2000).

In the first sticky mittens study, Needham et al. (2002) assessed visual looking behavior post‐test by presenting infants with two tasks. In the first task, four toys from the training were placed in front of the infant for 4 min, and in the second task four different and novel toys were placed in the infant's hand one at a time for 30–60 s. When presented with familiar toys, infants from the sticky mittens condition looked at these toys for 68 s, whereas infants from the no training condition looked at the same toys for only 33 s. However, as looking behavior was tested only post‐test in both conditions, we cannot assess whether an improvement in visual attention occurred and whether this was due to training. Furthermore, when presented with novel toys, there were no differences in looking between infants from the sticky mittens and no training conditions. In the shorter 9‐min sticky mittens study (Needham et al., 2017), a novel object was held in front of the infant for 1 min pre‐ and post‐test. An effect on visual attention to a novel object was demonstrated; looking times in the sticky mittens condition increased from 29 to 42 s, whereas infants in the observational condition had a decreased looking time from 29 to 21 s.

In an eye tracking study (Libertus & Needham, 2011) relying on the often‐used sample from the sticky mittens, observational, and no training conditions from Libertus and Needham (2010), pictures of faces and toys were shown side‐by‐side. After sticky mittens training, there was an effect on visual attention to faces. Infants from the sticky mittens condition looked at faces for 25% of the time post‐test, whereas the observational and no training conditions only looked at faces for 5% and 3% of the time, respectively. This interesting finding contradicts earlier discussed ideas that motor experiences may influence visual attention to objects. Libertus and Needham (2011) speculated that sticky mittens training may help infants understand the social importance of faces in the world through understanding that they and other people are self‐acting agents, and through a trigger of the motor resonance system that allows infants to understand other people's actions. Interestingly though, other studies find that infants in sticky mittens conditions either do not look more at the experimenter after training (Williams et al., 2015) or they show decreased looking at the experimenter (Libertus & Needham, 2010).

As reviewed earlier, Libertus et al. (2016) followed up on the infants who were part of the Libertus and Needham (2010) study. Fifteen‐month‐old infants from the sticky mittens condition looked at the object 76% of the time, whereas infants from the observational condition only did this for 55% of the time and the separate no‐training group only 65% of the time. This longitudinal study claims that motor abilities trained early in life using sticky mittens not only impact motor abilities later in infancy, but there may also be later emerging effects on visual attentional abilities. However, as discussed before, the sticky mittens condition had higher numerical baseline reaching, which may have independently influenced visual attention in the period between training and the 15‐month follow‐up.

Other sticky mittens work has demonstrated that infants do not change their visual attention directly after training. Libertus and Needham (2010) and Wiesen et al. (2016) did not demonstrate any differences in how long infants attended to objects pre‐and‐post‐test. Wiesen et al. (2016) demonstrated a tentative enhancement in object‐directed visual attention 2 months after the training. When infants held the toy in their hand, a small effect was found for infants in the sticky mittens condition; they looked at the toy 14 s post‐test and 23 s at follow‐up, whereas infants from the observational condition looked at the toy for 14 s on both occasions. Notably, these effects were only marginally significant. Libertus and Needham (2014) did not demonstrate enhanced visual attention to objects or faces in the sticky mittens condition. Only when infants acted on toys without encouragement (movement‐only condition), visual attention to faces increased, an effect that was not present with the other conditions. Williams et al. (2015) also found different looking outcomes compared to Needham et al. (2002). Infants in the sticky mittens condition looked at objects for 26% of the time, whereas infants in the non‐sticky mittens looked at objects for 46% of the time. Rather, sticky mittens looking behavior was characterized by more looks to the surroundings, which were 51% of the time compared to 36% in the non‐sticky mittens condition.

#### 3.3.2 Visual processing of objects

Some studies have begun to investigate whether mittens training affects the processing of more complex visual information. Rakison and Krogh (2012) investigated whether 4.5‐month‐old infants are able to perceive causality in actions. Two groups of infants wore mittens while they interacted with toys (either Velcro toys for sticky mittens, or normal toys for non‐sticky mittens). In a causal habituation task, infants saw a red ball moving forward, touching a green ball, and subsequently continuing forward. Infants’ perception of a reversed causal movement was tested. After sticky mittens training, infants were able to detect the causality whereas the infants in the non‐sticky mittens condition were not. The reaching experience was suggested to have provided infants with information about causality. However, further examination of this effect showed that infants were not able to generalize this newly acquired ability to detect simple causality in novel situations. As Gerson and Woodward (2014b) found, the effects of sticky mittens seemed to be context‐specific.

A more recent study was interested in whether the motor abilities of 4‐month‐old infants trained with sticky mittens impacted the infants’ ability to mentally rotate objects. Infants took part in the training either before or after the mental rotation assessment. In a habituation task, objects similar to those in the training would rotate. In test trials, new objects were mirrored and rotated. There was no effect of enhanced mental rotation abilities after reaching training. However, infants who trained before assessment exhibited more looking and touching behaviors during training compared to infants who trained after assessment. These visual and manual behaviors in the former condition were found to correlate with the ability to mentally rotate objects (Slone et al., 2018).

Overall, there is weak support for visual attention effects. Only one study has shown that infants look longer at novel objects post‐test (Needham et al., 2017). Five studies have indicated different results; infants look at objects when familiar objects are used, but not novel objects (Needham et al., 2002), infants do not show an increase in visual attention to objects (Libertus & Needham, 2010; Wiesen et al., 2016; Williams et al., 2015), and infants look more at faces than objects (Libertus & Needham, 2011). Although there are effects 1 year after training, these may not be due to the training, as these infants demonstrated inflated reaching prior to training at 3 months of age. To date, evidence does not support the notion that sticky mittens training enhances visual attention. Preliminary evidence suggests that effects on visual processing, causality, and mental rotation are interesting avenues for future work. In the next section, we will review the latest addition to the sticky mittens literature, the application of sticky mittens training to improve motor skills in developmental disorders associated with motor impairments.

### 3.4 Developmental disorders

Several researchers have started to investigate how this training can be applied to populations in which motor deficits are prevalent. Some developmental disorders, such as ASD (Libertus et al., 2014) and preterm children (van Haastert et al., 2006), have often been characterized by some type of delay in motor development. Infants with siblings who suffer from ASD often have higher chances of developing ASD themselves. At 3 and 6 months of age, the majority of these high‐risk infants exhibited motor delays, which were related to social impairments in their second year (Bhat et al., 2012). Notably, 6‐month‐old infants at high risk of ASD exhibited difficulties grasping objects (Ekberg et al., 2016; Libertus et al., 2014). Preterm infants also tend to exhibit delays in motor development in their first 2 years of life (van Haastert et al., 2006), and between the ages of 30 and 48 months infants exhibit lower cognitive functioning than children not born preterm (Caravale et al., 2005; Kaul et al., 2019). Another cause of motor impairment is cerebral palsy, which is often characterized by poor grasping abilities in the affected arm (Rönnqvist & Rösblad, 2007; Steenbergen et al., 2000).

The practice of providing children with Velcro loop mittens and allowing them to act on Velcro‐covered toys dates back to at least the 1980 s, two decades before the first sticky mittens study was published by Needham et al. (2002). These early interventions have focused on reducing motor impairment in children with severe motor problems, such as children with cerebral palsy. Caregivers were advised to attach Velcro hook strips onto toys and let their children touch and pick them up with Velcro loop mittens, often as part of larger training batteries in which different strategies are used to facilitate the use of limbs at the need of additional training (Musselwhite, 1986). Early on, this method was expected to have positive effects on gross motor attainment (Chadwick & Clark, 1980; Doctoroff, 2001; Klauber, 1996). Hsieh (2008) examined whether these adaptive Velcro toys (sticky mittens in combination with other training routines) benefitted preschoolers with severe motor impairment. The training as a whole elicited more toy manipulation than regular toys, but the isolated effect of sticky mittens is not known. More recently, Chorna et al. (2015) pre‐registered a randomized controlled trial in which 12‐ to 24‐month‐old infants with cerebral palsy were subjected to the 2‐week sticky mittens training as described in previous sections, but the results have yet to be published. Although there is not much research on the benefits of Velcro toys and mittens for cerebral palsy, for a long time this tool has been considered useful for enhancing the motor abilities of children with motor disorders (e.g., Doctoroff, 2001; Musselwhite, 1986).

Libertus and Landa (2014) conducted a study in which the effects of mittens training were examined in infants at risk of developing ASD. Three‐month‐old siblings of children with ASD were subjected to the sticky mittens training for 2 weeks. In contrast to previous studies, the experimenter placed the rattle within the infant's reach for 60 s. To test the benefits of the sticky mittens training for high‐risk infants, Libertus and Landa (2014) re‐used data from previous studies of typically developing infants (i.e., Libertus & Needham, 2010, 2014) as comparison groups. The high‐risk infants increased the time they engaged in reaching and grasping for a toy from 13% pre‐test to 31% post‐test, which was roughly equal to the typically developing infants in the sticky mittens condition reported by Libertus and Needham (2010). The high‐risk infants increased their reaching and grasping time more than infants from the observational, movement‐only, and encouragement‐only condition. However, the high‐risk infants exhibited a higher tendency for reaching prior to training, just as the regular sticky mittens condition did in Libertus and Needham (2010). In the same study (Libertus & Landa, 2014), sticky mittens training did not affect visual attention to toys and faces in a high‐risk group. According to the authors, the risk group enhanced their motor skills, but the effect was not strong enough to change high‐risk infants’ visual attention skills.

Similar effects of the sticky mittens training have been found for infants who were born premature. Five‐month‐old premature infants were assigned to either a 4‐min sticky mittens or a no‐training condition. To provide infants with more haptic feedback and gather more information from the training, the top parts of the mittens were cut off so the fingers were exposed. This training deviated slightly in that infants took part in the training in a supine position on the experimenter's lap. The experimenter moved toys to the infants’ midline, and then subsequently moved one of their hands toward the toy. After the infant interacted with the toy, the experimenter stroked the infants’ limb with the toy. Pre‐test, immediately after training (post‐test), and 4 min after training (follow‐up), a toy was held in front of the infant for 2 min. Post‐test, the sticky mittens condition resulted in a larger increase in the number of reaches compared to the no‐training condition. Although the number of reaches decreased at the follow‐up assessment, the sticky mittens training was considered possibly beneficial for infants who typically have motor delays (Nascimento et al., 2019). However, as the number of reaches decreased when assessed at follow‐up, more research is needed before interventions targeting motor delays in premature infants can incorporate mittens training. Similar facilitating effects were found when preterm infants received a reaching intervention that consisted of three combined trainings, of which one was a sticky mittens training. After training, infants in this combined motor training contacted the toy approximately 62 times and spent 43% of the time in contact with the toy, whereas infants in a control training (social) contacted the toy 22 times and contacted the toy for 16% of the time (Heathcock et al., 2008). However, in the combined motor training, the effects of the sticky mittens training itself are difficult to disentangle from the other motor trainings and thus difficult to evaluate.

In summary, sticky mittens have been used in cerebral palsy training for many years and often as part of larger training packages (Musselwhite, 1986), but the unique contribution of this training component remains unclear. More recently, evidence suggested that premature infants seem to exhibit more reaching behaviors post‐test (Nascimento et al., 2019). The effects on infants at risk for ASD are not clear, largely due to methodological problems and the re‐use of conditions as noted in several places throughout this review. More work is needed before a firm conclusion can be reached. Next, we will discuss what mechanisms underlying the sticky mittens training have been suggested until now.

## 4 HOW DO THE STICKY MITTENS WORK CONCEPTUALLY?

As is the case in all fields of research, the authors disagree on the mechanisms underlying the sticky mittens effect. In this section, we will review the different suggestions that have been brought forward in the current sticky mittens literature and the empirical evidence supporting each explanation, temporarily leaving behind any doubts about the validity of particular sticky mittens effects. The explanations are structured using the same outline as above, starting with explanations that target motor performance.

### 4.1 Motor explanations

The first explanation regarding the mechanisms responsible for the effect of sticky mittens was provided by Needham et al. (2002), attributing the effects to contingencies picked up in the training (*contingency learning*). As infants bring their hand to the toy, they observe the consequence of their own action upon contact, as the object sticks to the mitten, allowing the infants to manipulate it. They can now bring the toy closer to their eyes or bang the toy on the table. However, note that Williams et al. (2015) found no increased visual engagement with the object during each training session. Although a number of sticky mittens studies have been conducted since the original study, there have only been three studies that experimentally examined contingency learning in this particular context. Although contingency in the form of parental encouragement (Libertus & Needham, 2014) did not influence reaching, Needham et al. (2017) showed that 4‐month‐old infants responded differently to different levels of contingency in the sticky mittens training. They manipulated the amount of auditory feedback infants could perceive such that half of the infants would interact with louder than normal toys and the other half interacted with softer toys. The louder toys were found to induce longer periods of touching and looking at the toys. Another important source of contingency is haptic feedback (Williams & Corbetta, 2016), which is absent in the sticky mittens training and thus prevents naturalistic reaching development. Although inducing haptic feedback in the sticky mittens training was not enough to increase manual behaviors (Williams et al., 2015), the types of contingency that are picked up in the training may be limited yet enough to elicit reaching and grasping.

As another potential mechanism, Libertus and Needham (2014) proposed an *effectance motivation explanation*, in which the predictable consequences infants observe in the training help them develop enhanced motivation to keep performing reaching actions to obtain the object. Currently, the sticky mittens studies do not allow us to examine an increased motivation to act on objects. More recently, it was proposed that the sticky mittens training promote integration of different types of sensory modalities. During training, infants encode information from visual, auditory, manual, and proprioceptive modalities, which create redundant information about one specific object. This *intersensory redundancy explanation* suggests that, in turn, more effective manual and visual exploration abilities are promoted (Needham et al., 2017; Williams & Corbetta, 2016; Williams et al., 2015).

### 4.2 Social perception explanations

Supporters of *proprioceptive* explanations have proposed that social cognitive effects come from agentive experiences infants gain during training. In the training, infants independently act on objects and learn to perceive themselves as an intentional agent that wants to attain a goal. Restructuring their representations, they learn to perceive others as active agents who also want to attain a goal (Gerson & Woodward, 2014a, 2014b; Libertus & Needham, 2014; Sommerville et al., 2005). In turn, these developments lead to more opportunities for triadic interactions. As infants experience reaching and grasping through mittens training, they may try to engage their caregiver in social exchanges whereby the infant looks at the toy and then to the caregiver's face (Libertus & Landa, 2014; Needham, 2016).

Unlike other explanations of what infants learn during training, Skerry et al. (2013) do not believe infants learn about efficient reaching through training. Instead, they relate their findings to the *teleological stance* explanation (Gergely & Csibra, 2003). Infants are thought to already possess abstract knowledge of how reaches are performed efficiently, but they cannot access this information yet because they are unable to identify other people's goals while reaching. When they learn to reach via training, they learn to encode the goals of such actions, which give them access to knowledge of whether the goal is obtained efficiently (Skerry et al., 2013). The explanation proposed by Skerry et al. (2013) is more of a nativist statement, whereas others have proposed a more diverse set of explanations.

More recently, Liu et al. (2019) extended the teleological stance of Skerry et al. (2013) such that the abstract knowledge of efficiency is suggested to be learned from observing others in the first few months prior to reaching emergence, with a possibility of this process starting before birth. This idea is consistent with the explanation Juvrud and Gredebäck (2020) proposed that integrates infants’ own action experiences with their understanding of efficient reaches, which is referred to as the *embodied theory of teleological processes*. When infants are learning to reach for objects, they learn first‐hand about efficient reaching by observing deviations in the path they take to the object relative to the efficient and straight path. This process may start as early as in the womb. Active experience and observing these deviations during training rather than abstract knowledge may bring about this understanding of efficiency.

Several researchers have put forward a neurological explanation for the social‐cognitive effects of the sticky mittens, namely a *motor resonance* explanation. The social‐cognitive effects may come about through activation of the mirror neuron system that is active not only when infants produce an action, but also when they observe an action. With respect to the sticky mittens training, this would entail the simulated reaching experiences activating the neural systems responsible for representations of self‐produced actions, which in turn become activated when observing others (Gerson & Woodward, 2014a, 2014b; Libertus & Needham, 2011; Rakison & Krogh, 2012).

### 4.3 Visual attentional explanations

The sticky mittens have also been suggested to impact visual attention by opening up a great deal of learning opportunities about objects. This *active exploration* explanation entails that being able to pick up a toy with a mitten enables infants to inspect that toy visually or orally in more detail, which enables them to learn about specific object properties (Libertus et al., 2016; Needham et al., 2002; Wiesen et al., 2016). The training could also open up opportunities to observe objects from multiple angles, although it did not directly influence mental rotation abilities (Slone et al., 2018).

The ability of infants to bring objects closer to their eyes taps into a related explanation of the training's effect, which we refer to here as the *redistribution of attention* explanation. The training may induce a redistribution of attention whereby infants attend more to their training toys and their hands. This may subsequently transfer to infants’ looking behaviors during (visual attention or social perception) assessments. They may attend more to certain objects, such as the goal of a person performing a reach or a ball that causally contacts another ball (Bakker et al., 2016; Rakison & Krogh, 2012; Sommerville et al., 2005). Overall, it is clear that researchers have brought forward many useful suggestions for why the sticky mittens exert their effects. These suggestions offer us many starting points for future research.

## 5 CONCLUSION

Initially, the sticky mitten training was set‐up to improve manual behaviors in 3‐month‐old infants. Having infants act on Velcro‐covered objects while wearing mittens affected the levels of reaching and grasping in infants who had not begun to do this (Needham et al., 2002). Although there is some evidence that this training could potentially improve reaching and grasping behaviors, much more work is needed before we can make firm statements about the effects in the motor domain. Seeing as this training shows robust effects in the social perception domain, it is important that we investigate what lies behind these incredible effects and whether or not these effects are elicited via motor improvements. Future studies need to take the shortcomings, discussed earlier, into account when designing new studies along with preregistering these studies to get a clearer picture of motor improvements. The sticky mittens studies to date have reported inconsistent findings, with two studies showing immediate effects of training (Libertus & Needham, 2010; Needham et al., 2002), one study finding only a delayed effect 2 months after training (Wiesen et al., 2016), two studies finding no effects at all (Needham et al., 2017; Williams et al., 2015), and one study finding long‐term improvements (Libertus et al., 2016).

Although some work provides tentative evidence that sticky mittens training may exert motor effects via contingency learning, firm statements cannot be made. Although sensory feedback is important for the natural progression of reaching and grasping (Williams & Corbetta, 2016), it remains unclear what the role of contingency learning is in the manual effects of the sticky mittens training and how this comes into play in the secondary effects discussed earlier. More work will be necessary to examine this explanation, or what other explanation may be better suited.

Thus, the manual effects seem to be more complicated than the social perceptual effects typically associated with motor maturity. Inevitably, this raises the question of whether sticky mittens training in its current form is powerful enough to enhance these manual behaviors. As is the case for other motor skills in infancy (Adolph et al., 2012), the development of reaching is characterized as a protracted process with extensive practice before a motor skill emerges (Corbetta & Snapp‐Childs, 2009; Konczak & Dichgans, 1997). Reaching onset typically occurs around 4–6 months of age (von Hofsten, 1984), whereas sticky mittens training begins at 3 months of age. Given that infants require massive motor practice before motor skills emerge, it seems unlikely that the natural development between 3 and 6 months of age can be reduced to only 140 min of reaching training using sticky mittens. Perhaps this rather short training lacks the strength to cause strong motor patterns. Although learning to reach and grasp involves a larger set of actions (Bertenthal & von Hofsten, 1998) and sensory feedback (Corbetta et al., 2016; Williams & Corbetta, 2016), we may be required to investigate the effects of longer and more intensive sticky mittens training. However, caution is needed in designing these studies, as the effects of longer sticky mittens training are unknown. In parallel, training sessions closer to the length of the Needham et al. (2002) training may possibly be necessary to inform us whether the secondary social perceptive effects hold true, as currently the results are based solely on a single experience with sticky mittens.

The lack of replication regarding reaching and grasping behaviors may also stem from the fashion in which these behaviors are measured. As reaching and grasping are two different (though functionally and temporally connected) behaviors (Karl & Whishaw, 2013; Paulignan et al., 1990; Rouse et al., 2018) and different measures are used in the sticky mittens studies, it is not apparent which of these behaviors the training enhances. Furthermore, in the majority of these studies, manual behaviors were operationalized in terms of latency, whereas only one study examined reaching in terms of quantity (Nascimento et al., 2019). Perhaps with more consistent and appropriate measures, more consistent effects on reaching and grasping will be found. Future independent studies may need to consider collecting kinematic reaching and grasping data, such as in Williams et al. (2015). Reach may be operationalized as the number of reaches, the smoothness of the reach, or the number of movement units comprising the reach (von Hofsten, 1991), whereas grasp may be defined as the time when a grasp is initiated or when in time the fingers curl around the target (von Hofsten & Rönnqvist, 1988). Given the complexity of these effects and inconsistencies in methodological choices, future work may also benefit from pre‐registering sticky mittens studies.

Regarding the social perception effects of sticky mittens training, clear and robust effects have been found. In general, we can conclude that mittens training change what infants attend to in a social context. This newfound understanding of actions shows the incredible potential sticky mittens have in influencing social cognitive development in the first year of life. Although some studies have indicated that these social perception effects do not generalize to other contexts, we should keep in mind that the social perception effects were found after extremely short training sessions. Future work in the social‐cognitive domain will likely benefit strongly from investigating whether longer sticky mittens training has robust action‐understanding effects, and whether these effects generalize to different situations.

Although the social‐cognitive effect from sticky mittens is clear, one critical question has not been addressed by any of the action‐perception studies. One explanation for action understanding is that infants who have experience performing actions through these actions are able to understand other people's actions (e.g., Gredebäck & Falck‐Ytter, 2015). Sticky mittens training was brought forward in this domain as a paradigm that can help us understand this correlational relationship (Sommerville et al., 2005). However, this review makes it clear that the studies on manual behaviors do not show robust effects of sticky mittens training. If there is a possibility that infants are not learning new motor skills, how are improvements in the social‐cognitive domain explained? Two studies have attempted to explain this as an innate “core knowledge” teleological stance (Skerry et al., 2013) or an experience‐dependent process based on manual actions before testing (Juvrud & Gredebäck, 2020). Without additional data, these two explanations are difficult to separate.

Although the social perception results seem more likely to be robust, the visual attentional studies show the opposite results. The sticky mittens work examining visual attention has found differing effects. It is currently unclear if there is any relationship between reaching training and visual attentional abilities and, if there is a connection, whether there is enhanced attention to objects or faces. Although prior work indicated that the natural onset of reaching and grasping directed visual attention toward objects (Corbetta et al., 2014), the training does not always seem to elicit these effects. Consequently, this complicates our search for a fitting theory underlying the sticky mittens training. In order to understand this relationship, more work is needed to investigate whether infants go through a redistribution of attention or are capable of inspecting objects in more detail during and after training.

## Supporting information

Supplementary Information

## Data Availability

Data sharing is not applicable to this article as no new data were created or analyzed in this study.

## ENDNOTES

^1^
Reaching and grasping are discussed as one measure unless stated otherwise.

^2^
In this study and all others outlined in this section, an experimenter interacted with the infant rather than a parent, in contrast to the protocol used in the studies of manual reaching.
