# Supplementary material for: The sticky mittens paradigm: A critical appraisal of current results and explanations
Source: Dev Sci. 2020 Oct 5;24(5):e13036. doi: 10.1111/desc.13036 (PMC8518992; doi:10.1111/desc.13036)
Supplement: Supplementary file 1 — Supplementary Information [file DESC-24-e13036-s001.docx]

**Supplementary Information**

**Sticky Mittens Methodology**

In this section, we will give an overview of important methodological choices made in the sticky mittens literature (see Table S1 for an overview). Age ranges in the sticky mittens literature have been rather consistent. All studies report age ranges that lie beneath the typical age of reaching emergence (i.e., 5 to 6 months, von Hofsten, 1984; von Hofsten & Rönnqvist, 1988). Whereas the motor development studies have chosen to investigate the sticky mittens in infants aged 2-to-3 months, the social perception studies typically investigate sticky mittens effects at the age of 4 months.

All sticky mittens findings are contrasted against control groups. Typically, studies compare these findings against a no training control group but also a control group in which infants take part in a non-reaching training. In the latter control groups, some infants are exposed to situation in which they can try to reach for an object without it sticking to the mitten, whereas other groups only provide infants with the opportunity to observe someone else reaching for the object for them. A divide in the length of training is clearly visible between motor development and social perception studies, with the former studies typically requiring a 2-week training whereas the latter studies employ only single training sessions of several minutes.

Whether the sticky mittens training is facilitated by the parent or experimenter appears to relate to whether the study regards motor development or social perception. Motor development studies typically have the parent conduct the training, although the experimenter conducts the training in Needham et al. (2017) and Williams et al. (2015). In Social perception studies, the experimenter always conducts the training. Regarding the amount of encouragement during training, it is unclear in most studies whether this is part of training. In motor development studies, parents typically encourage infants to swat toys, except for Williams et al. (2015) or in the case of a no encouragement control (Libertus & Needham, 2014). In other studies however, it is unclear what role encouragement has played in training infants.

More differences in methodology can be seen when the time of assessment is considered. Most motor development studies assess manual behaviours before and after training. Moreover, two studies conducted follow-up assessments two months (Wiesen et al., 2016) and 12 months (Libertus et al., 2016) after training. In (social) perception studies, a few studies only assess their target behaviours after training, although most studies conduct pre-and post-testing. In the motor development studies, most studies use novel toys for posttest assessments. In the (social) perception studies, toys from training are often used, though in different forms (e.g., habituation paradigms). Two common reaching assessments within motor development studies are the 4-step-reaching procedure with incrementing toy placement (e.g., Libertus & Needham, 2010) and a task in which a toy is placed at midline (e.g., Williams et al., 2016). Attentional effects of sticky mittens have been examined through face preference tasks in which toy-face pairs are presented to the infant (e.g., Libertus & Needham, 2011). Other studies use visual habituation paradigms with reaching movements (e.g., Sommerville et al., 2005) for social perception development or rotating objects (Slone et al., 2018) and casual events (Rakison & Krogh, 2012) for perceptual development.

**Table 1**

*Overview of Sticky Mittens Methodology.*

| Study | N | Conditions | Age | Type of Mittens | Training Duration | Conducted by | Encouragement | Time of Assessment | Toys at Assessment | Assessment |
| --- | --- | --- | --- | --- | --- | --- | --- | --- | --- | --- |
| Bakker et al., 2016 | 30 | Sticky mittens (n = 15)  Observational ( n = 15) | 4 | Full-hand Velcro | 4 min | Experimenter | Yes | Posttest | -- | Electroencephalography recording of (in)congruent grasps |
| Gerson & Woodward, 2014a | 72 | Sticky mittens (n = 24)  Observational (n = 24)  No training (n = 24) | 3.5 | Full-hand Velcro | 3 min | Experimenter | Unclear | Pretest  Posttest | Familiar | Visual habituation |
| Gerson & Woodward, 2014a | 90 | Sticky mittens (n = 30)  Observational (n = 30)  Second sticky mittens (n = 30) | 3.5 | Full-hand Velcro | 3 min | Experimenter | Unclear | Pretest  Posttest | Familiar | Visual habituation |
| Libertus & Landa, 2014 | 89 | High risk autism spectrum sticky mittens (n=17)  Sticky mittens (n = 18) ^b^  Observational (n = 18) ^b^  Encouragement (n = 18) ^c^  Movement (n = 18) ^c^ | 2 – 3 | Full-hand Velcro | 14 days, 10 minutes a day | Parent | Yes | Pretest  Posttest | New | Toy placed on table  Face preference |
| Libertus & Needham, 2010 | 58 | Sticky mittens (n = 18)  Observational training (n= 18)  No training 3 months (n= 19)  No training 5 months (n = 23) | 2 - 3 | Full-hand Velcro | 14 days, 10 minutes a day | Parent | Yes | Pretest  Posttest | New | 4-step-reaching |
| Libertus & Needham, 2011 | 55 | Sticky mittens (n = 18) ^b^  Observational training (n= 18) ^b^  No training 3 months (n= 19) ^b^  No training 5 months (n = 23) ^b^ | 3 | Full-hand Velcro | 14 days, 10 minutes a day | Parent | Yes | Posttest | New | 4-step-reaching  Face preference |
| Libertus & Needham, 2014 | 72 | Sticky mittens (n = 18) ^b^  Observational training (n= 18) ^b^  Encouragement (n= 18)  Movement (n = 18) | 2 - 3 | Full-hand Velcro | 14 days, 10 minutes a day | Parent | Yes | Pretest  Posttest | New | 4-step-reaching  Face preference |
| Libertus et al., 2016 ^a^ | 40 | Sticky mittens (n = 14)  Observational training (n= 11)  No training (n= 15) | 15 | -- | -- | -- | -- | 12-month follow-up | New | 5-minute free play |
| Nascimento et al., 2019 | 24 | Sticky mittens (n = 12)  No training (n =12) | 4^d^ | Velcro mittens with opening for thumb and fingers | 4 minutes | Experimenter | Unclear | Pretest  Post test  2 minutes post test | Familiar | Toy held at midline |
| Needham et al., 2002 | 32 | Sticky mittens (n = 16)  Observational training (n= 16) | 3 | Full-hand Velcro | 14 days, 10 minutes a day | Parent | Yes | Posttest | Familiar and new | Toy held at midline |
| Needham et al., 2017 | 38 | Sticky mittens (n = 19)  Observational (n = 19= | 4 | Full-hand Velcro | 9 minutes | Experimenter | Yes | Pretest  Posttest | New | Toy held at midline |
| Rakison & Krogh, 2012 | 40 | Sticky mittens (n = 20)  Non sticky mittens (n = 20) | 4 | Full-hand Velcro | 3 minutes | Experimenter | Unclear | Posttest | Familiar and new | Visual habituation |
| Skerry et al., 2013 | 112 | Sticky mittens ( n = 20)  Non sticky mittens ( n= 20)  No training (n = 20)  Constrained sticky mittens (n = 26)  Unconstrained sticky mittens (n = 26) | 3 – 4 | Full-hand Velcro | 3 minutes | Experimenter | Unclear | Posttest | Familiar | Visual habituation |
| Slone et al., 2018 | 80 | Sticky mittens (n = 40)  No sticky mittens (n = 40) | 4 | Full-hand Velcro | 4 minutes | Experimenter | Unclear | Post test | Familiar | Visual habituation |
| Sommerville et al., 2005 | 30 | Sticky mittens ( n= 15)  No training (n = 15) | 3 | Full-hand Velcro | 3.5 minutes | Experimenter | Unclear | Pretest  Post test | Familiar | Visual habituation |
| Wiesen et al., 2016 | 32 | Sticky mittens (n = 16)  Observational (n = 16) | 2 – 3 | Full-hand Velcro | 14 days, 10 minutes a day | Parent | Yes | Pretest  Posttest  2 months posttest | New | Toy held at midline  4-step-reaching |
| Williams et al., 2015 | 37 | Sticky mittens (n = 13)  Non sticky mittens (n = 11)  No training (n = 13) | 2 – 3 | Velcro mittens with opening for thumb and fingers | 14 days,  10 minutes a day | Experimenter | No | Pretest  Posttest | New | Toy held at midline |

*Note. Total number of participants and number of participants in each condition are reported. Age is reported in months.*

^a^ This study was a follow-up of Libertus & Needham (2010).

^b^ These conditions were taken from Libertus & Needham (2010).

^c^ These conditions were taken from Libertus & Needham (2014).

^d^ Twelve weeks corrected age.
